# Pharmacokinetics of Intravenous, Intramuscular, Oral, and Transdermal Administration of Flunixin Meglumine in Pre-wean Piglets

**DOI:** 10.3389/fvets.2020.00586

**Published:** 2020-08-28

**Authors:** Heather C. Kittrell, Jonathan P. Mochel, Justin T. Brown, Anna Marie K. Forseth, Kristen P. Hayman, Suzanne M. Rajewski, Johann F. Coetzee, Benjamin K. Schneider, Brette Ratliffe, Kristin J. Skoland, Locke A. Karriker

**Affiliations:** ^1^Swine Medicine Education Center, College of Veterinary Medicine, Iowa State University, Ames, IA, United States; ^2^SMART Pharmacology, Department of Biomedical Sciences, College of Veterinary Medicine, Iowa State University, Ames, IA, United States; ^3^Animal Health Division, Montana Department of Livestock, Helena, MT, United States; ^4^Analytical Chemistry Services, College of Veterinary Medicine, Iowa State University, Ames, IA, United States; ^5^Department of Anatomy and Physiology, College of Veterinary Medicine, Kansas State University, Manhattan, KS, United States

**Keywords:** swine, non-steroidal anti-inflammatory, flunixin meglumine, pain, topical, NLME

## Abstract

Castration and tail-docking of pre-wean piglets are common procedures that are known to induce pain and would benefit from pain mitigation. Flunixin meglumine (FM) is a non-steroidal anti-inflammatory drug currently approved in the United States for pyrexia in swine and lameness pain in cattle. The objective of this study was to establish the pharmacokinetic (PK) parameters resulting from intravenous (IV), intramuscular (IM), oral (PO) and transdermal (TD) administration of FM in pre-wean piglets. FM was administered to thirty-nine pre-wean piglets at a target dose of 2.2 mg/kg for IV and IM and 3.3 mg/kg for PO and TD route. Plasma was collected at twenty-seven time points from 0 to 9 days after FM administration and concentrations were determined using ultra-high performance liquid chromatography coupled with mass spectrometry (UPLC-MS). Pharmacokinetic data were analyzed using noncompartmental analysis (NCA) methods and nonlinear mixed-effects (NLME). Initial plasma concentration for IV (C_0_) 11,653 μg/L and mean peak plasma concentrations (C_max_) 6,543 μg/L (IM), 4,883 μg/L (PO), and 31.5 μg/L (TD) were measured. The time points of peak FM concentrations (t_max_) were estimated 30 min, 1 h, and 24 h for IM, PO, and TD, respectively. The bioavailability (*F*) of PO and IM FM was estimated at >99%, while the bioavailability of TD FM was estimated to be 7.8%. The reported C_max_ of FM after IM and PO administration is consistent with therapeutic concentration ranges that mitigate pain in other species and adult pigs. However, the low estimated concentration of FM after TD dosing is not expected to mitigate pain in pre-wean piglets. The low *F* of TD FM suggests that expanding the surface area of application is unlikely to be sufficient to establish an effective TD dose for pain, while the high bioavailability for PO FM should allow for an effective dose regimen to be established.

## Introduction

Consumers in the United States (US) view animal welfare as the most important characteristic of an “ideal pig/pork farm” (1). Respondents of a survey indicated that physical and emotional comfort is a primary aspect of animal welfare and desired that “piglets wouldn't be castrated or teeth clipped without some form of pain killer” (1). Castration and tail-docking are considered painful procedures and are routinely performed on pre-wean piglets throughout the US (2). Flunixin meglumine (FM) is a non-steroidal anti-inflammatory drug (NSAID) with non-selective cyclooxygenase (COX) inhibitory action (3). Inhibition of COX-2 reduces prostaglandin production responsible for inflammation, pyrexia, and pain. FM is approved for the control of pyrexia associated with swine respiratory disease in swine, and pyrexia and inflammation in cattle in the US. In 2017, a transdermal (TD) application of FM was approved for foot rot pain in cattle and pyrexia associated with bovine respiratory disease (4). Although FM is not approved for pain control in pigs, Animal Medicinal Drug Use Clarification Act (AMDUCA) algorithms suggest that the product should be considered when treating pain in food animal species (5). Administration routes that do not require individual piglet injection would be preferable for on-farm use due to worker safety, infection control, and piglet welfare. With the TD formulation now commercially available, there is potential for on-farm application during pre-weaning processing in swine; however, we must confirm that FM achieves effective plasma concentrations and provide data to develop a dose regimen that is safe for the pre-wean piglets.

The objectives of this study were to establish the pharmacokinetics (PK) of IV, IM, PO, and TD administration of FM in pre-wean piglets, and evaluate the ability of TD FM to reach therapeutic levels.

## Materials and Methods

### Animals and Housing

Healthy, cross-bred, mixed-sex piglets, 9 days of age, with no previous FM treatment and an initial mean weight of 3.40 ± 0.58 kg were utilized in two phases of study. The piglets had received routine, on-farm processing at 3 days of age that included iron dextran injection and tail docking of all piglets, and castration of male piglets. Phase I consisted of a parallel design in which twenty-three piglets were assigned to either IM, PO, or TD administration groups for PK evaluation. Phase II consisted of sixteen piglets in a two-way crossover study of intravenous (IV) and TD administration of FM. Piglets were individually housed in a climate-controlled room and each pen had a heat lamp that provided supplemental heat to maintain a microenvironment temperature of 32–35°C. Room temperature was gradually decreased and supplemental heat was removed as size and age of pigs increased. An orogastric feeding tube (Sovereign™, CardinalHealth, Dublin, OH, USA) was used to provide nutrition (Esbilac® Pet-Ag, Inc, Hampshire, IL, USA) three times daily with *ad libitum* water (6). Phase I pigs were maintained on the Esbilac® diet for the entirety of the study. Phase II pigs were gradually weaned onto a dry starter ration by day 10 that met or exceeded recommended NRC guidelines (7). Feeding schedules were modified slightly to allow for blood collection time points. This study was approved by the Institutional Animal Care and Use Committee (IACUC # 18-057, # 18-169) at Iowa State University.

### Study Design-Phase I-PK of Oral, Transdermal, and Intramuscular Flunixin Meglumine

Piglets were randomly allotted to one of three treatment groups differing by route of administration: intramuscular (IM, *n* = 7), oral (PO, *n* = 8), and transdermal (TD, *n* = 8). A randomized block design ensured that an even number of males and females were assigned to each group. After group allocation, a pre-trial blood collection was performed on each piglet. One mortality resulted due to jugular vein hematoma, and therefore the intramuscular group consisted of 7 piglets. Flunixin meglumine was administered at a target dose of 2.2 mg/kg for IM (Banamine®-S, Merck Animal Health, Madison, NJ, USA) administration and a target dose of 3.3 mg/kg for PO and TD (Banamine®-S and Banamine® Transdermal, Merck Animal Health, Madison, NJ, USA). Piglets were weighed the day prior to drug administration to calculate drug dose for each pig. A single-use needle and syringe was used to administer FM in the musculature of the lateral neck, behind the ear. A new feeding tube was used for orogastric intubation and PO administration of FM, followed by a volume of water equal to the volume of the tube to ensure administration of the full dose. TD application consisted of a single-use syringe to apply FM to the dorsal midline skin between the shoulder blades.

### Study Design-Phase II- Bioavailability of Transdermal Flunixin Meglumine

A randomized block design was used to allocate pigs to one of two initial routes of administration: IV (*n* = 8) and TD (*n* = 8). IV administration was accomplished via jugular venipuncture. TD application was achieved as stated previously. Following treatments and blood collections, the groups were allowed a 9-day washout period, the routes of administration were switched for the groups, and sample collections were repeated.

### Blood Collection

Blood samples (1.0 mL/sample) were collected prior to treatment administration (0 min) and at 15, 30, 45, 60, 90 min and 2, 3, 6, 12, 24, 36, 48, 60, 72 h post treatment for all routes of administration in Phase I. Phase II blood collections occurred at 0, 5, 15, 30, 45, 60, 90 min and 2, 3, 6, 12, 24, 36, 48, 60 h post administration for the IV route and 0, 15, 30, 45, 60, 90 min and 2, 3, 6, 12, 24, 36, 48, 60, 72, 84, 96, 108, 120, 132, 144, 156, 168, 180, 192, 204, and 216 h for the TD route. The blood collection time points and washout period for TD application in this study were acquired from PK research conducted in cattle (8). All blood collections occurred via jugular venipuncture with a single use needle and syringe. Samples were immediately transferred to a sodium heparin blood collection tube (BD Vacutainer, Franklin Lakes, NJ, USA) and stored on ice before processing. Blood samples remained on ice for no longer than 2 h prior to centrifugation for 10 min at 1,500g. Collected plasma was placed in cryovials and frozen at −70°C until drug concentration analysis.

### UPLC-MS Analysis of Flunixin Meglumine Concentrations

Plasma concentrations of FM were determined using ultrahigh performance liquid chromatography (Q Exactive Focus Orbitrap, Thermo Scientific, San Jose, CA, USA) coupled with mass spectrometry (Dionex Ultimate 3000, Thermo Scientific, San Jose, CA, USA), as previously described (8). The standard curve for FM was prepared in blank porcine serum and ranged from 2 to 5,000 ng/mL with a correlation coefficient greater 0.995 for all analyses. Quality control (QC) samples were prepared at concentrations 30, 300, and 3,000 ng/mL. For concentrations above 3,000 ng/ml, dilution using blank swine serum was validated as a precise method for quantifying concentration. All dilutions in this study were <0.1. The QCs were ±15% of the nominal value. The lower limit of quantification (LLOQ) and lower limit of detection (LLOD) were 2 and 0.3 ng/mL, respectively. For Phase I, the accuracy and precision for the QC samples were 109 and 3.0% for the 30 ng/mL QC; 102 and 4.7% for the 300 ng/mL QC; and 105 and 4.2% for the 3,000 ng/mL QC. For Phase II, the accuracy and precision for the QC samples were 103 and 5.5% for the 30 ng/mL; 100 and 3.3% for the 300 ng/mL; and 104 and 4.8% for the 3,000 ng/mL samples.

### Noncompartmental Parameter Estimates

For completeness and to derive initial (i.e., starting) PK parameter values for the NLME model, we computed a complementary set of PK parameters via non-compartmental analysis (NCA). NCA was performed using PKanalix (Monolix Suite 2019R2, Lixoft, France). Where applicable, the following population summary as well as dosing group summary parameters were calculated area under plasma concentration-time curve/dose (AUC/Dose), (AUC_INF_), apparent clearance (Cl/F), clearance (Cl), maximum concentration (C_max_), terminal half-life (t_1/2_), terminal phase elimination rate constant (λ_z_), mean residence time (MRT), time of maximum concentration (t_max_), apparent volume of distribution at steady state (V_ss_/F), and volume of distribution at steady state (V_ss_).

### NLME Model Building and Evaluation

No outliers were identified during initial data exploration in Monolix datxplore (2019R2, Lixoft, France), allowing us to pool all data for model building. Flunixin meglumine plasma concentration time-courses from IV, IM, PO, and TD were analyzed simultaneously using the stochastic approximation expectation maximization algorithm (SAEM) as implemented in Monolix 2019R2 (Lixoft, France). Individual model parameters were obtained using the full posterior of the conditional distribution (9, 10). NLME models were written as previously described (9, 11–13).

Equation 1:

$$\begin{array}{l} y_{ij}=F\left(\phi_{i},\beta_{i},\;t_{ij}\right)+G\left(\phi_{i},\;{\;t}_{ij}\right)\cdot \varepsilon_{ij}\\ \varepsilon_{ij}\;\sim \;N\left(0,\sigma^{2}\right),\;\phi_{i}=h\left(\mu ,\eta_{i},\beta_{i}\right),\\ \eta_{ij}\;\sim \;N\left(0,\Omega ,\omega^{2}\right),\;j\in \left\{1,\;\ldots ,n_{i}\right\},\;i\in \left\{1,\ldots ,N\right\} \end{array}$$

*y*_*ij*_ is the observed FM concentration value for individual *i* at time *j*. *F(***φ**_*i*_, **β**_*i*_*, t*_*ij*_*)* is the model predicted value for the *i*^*th*^ individual at time *t*_*ij*_, with vector of individual parameters **φ**_*i*_ and vector of individual covariates **β**_*i*_.

*G(***φ**_*i*_*, t*_*ij*_*)* · ε_*ij*_ is the residual error model—a combination of both unexplained variability and measurement error. The function *G(***φ**_*i*_*, t*_*ij*_*)* is the scale of predicted error at time *t*_*ij*_, given the vector of individual parameters **φ**_*i*_. The values in vector **ε**_*ij*_ are distributed normally with mean **0** and variance σ^2^.

Individual parameters **φ**_*i*_ were modeled as a function, *h(***μ**, **η**_*i*_, **β**_*i*_*)*, of the mean individual parameter values, **μ**, individual variability **η**_*i*_, and individual covariates, **β**_*i*_. **η**_*i*_ are distributed normally with mean **0**, variance-covariance matrix **Ω**, and variance ω^2^. *j* is the index given to individual-level observations, taking values from 1 to *n*_*i*_. *i* is the index given to individuals, taking values from 1 to *N*.

Typically, individual parameters are modeled such that individual parameters **φ**_*i*_ are distributed log-normally,

Equation 2:

$$\begin{array}{l} \phi_{i}=\mu \cdot e^{\eta_{i}\;+\;\beta_{i}} \end{array}$$

However, TD and PO bioavailability were modeled such that values of bioavailability, **F**_*i*_, fall strictly between 0 and 1. This is achieved using the logit function as a link-function (i.e., **F**_*i*_ are distributed logit-normally). **ξ** is the vector of mean population bioavailability.

Equation 3:

$$\begin{array}{l} \log\left(\frac{{\text{F}}_{i}}{1\;-\;{\text{F}}_{i}}\right)=\log\left(\frac{\xi }{1\;-\;\xi }\right)+\eta_{i}+\beta_{i} \end{array}$$

### Handling of Data Below Limit of Quantification (BLQ)

Data below the lower limit of quantification (LLOQ) were modeled by adding a term to the likelihood function representing the probability that the true observation lies between zero and the LLOQ (10, 14, 15). For the calculation of the likelihood, this is equivalent to the M3 method implemented in NONMEM.

### Random Effects Correlation Estimates

Visual inspection of the scatterplot of random effects as well as Pearson correlation tests between random effects (threshold *p* < 0.05) were used to evaluate statistical correlations between model parameters. In agreement with previous literature (16), several samples of the posterior distribution obtained during the last iteration of the SAEM algorithm, rather than the empirical Bayes estimate (EBE), were used when producing the scatterplot to better assess correlation between model parameters.

### Inclusion of Covariate Relationships

Sex and bodyweight were tested for inclusion in the model using ANOVA and Pearson correlation test, respectively (threshold *p* < 0.05). Additionally, during the initial exploration of the individual time-course of FM PK, we noticed there appeared to be two distinct sub-populations of piglets. The first set of individuals appeared to be metabolizing FM relatively quickly and the second relatively slowly. After categorizing each individual as either a fast (MET = 0) or slow (MET = 1) metabolizer, we tested the classification for inclusion using the ANOVA method (threshold *p* < 0.05) as implemented in Monolix 2019R2.

### Model Evaluation

Convergence of the SAEM algorithm was assessed by inspection of the search stability of both the fixed and random effects parameter searches, as well as the stability of the log-likelihood estimate during parameter search. Standard goodness-of-fit plots, including individual predictions vs. observations, the distributions of weighted residuals (IWRES), and scatter plot of the residuals, were used to assess the performance of candidate models. Prediction distributions constructed from 500 Monte Carlo simulations were used to evaluate the ability of the final model to reproduce the variability in the observed PK data. Residual error estimates from the mathematical models were used as supportive information for evaluation of goodness-of-fit.

Normality and independence of residuals were assessed using histograms, quantile-quantile plots, and autocorrelation of conditional weighted residuals. For stable models with satisfactory goodness-of-fit diagnostics, final model selection was based on the Bayesian Information Criteria (BIC) as well as the relative standard error (RSE) of parameter estimates (a measure of the precision of the model parameter estimates). The BIC was selected over the Akaike Information Criterion as it tends to select simpler and more parsimonious models (17).

### Estimating a Therapeutic Topical Dose via Monte Carlo Simulations

After model selection and fit, we used Monte Carlo simulations to estimate the potential FM TD dosages at which FM would reach therapeutic plasma concentrations. To do this, we simulated the average plasma time-course of FM using the fit model (with inter-individual variability, but without error) at 1, 2, 4, 8, 14, and 20 mg/kg dosage. To derive the average plasma time-course at each dosage, we simulated 500 individuals for each dosage and computed the mean time-course from the simulated population.

Our metric of efficacy was the amount of time average FM plasma concentrations remained above one of 4 target pharmacodynamic targets (IC_50_ against COX-1: 21.46 ug/L, IC_50_ against COX-2: 63.34 ug/L, IC_80_ against COX-1: 390.52 ug/L, IC_80_ against COX-2: 894.63 ug/L) (18).

Monte Carlo simulations were performed in R 3.6.1 (The R Foundation for Statistical Computing) using the mlxR package (maintained by Lixoft, France).

## Results

### Animals

No noticeable signs of discomfort were observed after administration of FM. One pig regurgitated during administration of PO FM in Phase I, leading to an inability to determine the correct input dose and was therefore excluded from data modeling. During administration of FM in Phase II, we were unable to verify IV administration in two pigs and data points were removed that were inconsistent with expected IV plasma concentration-time curve (11 data points).

### Noncompartmental Parameter Estimates

**Figure 1:**
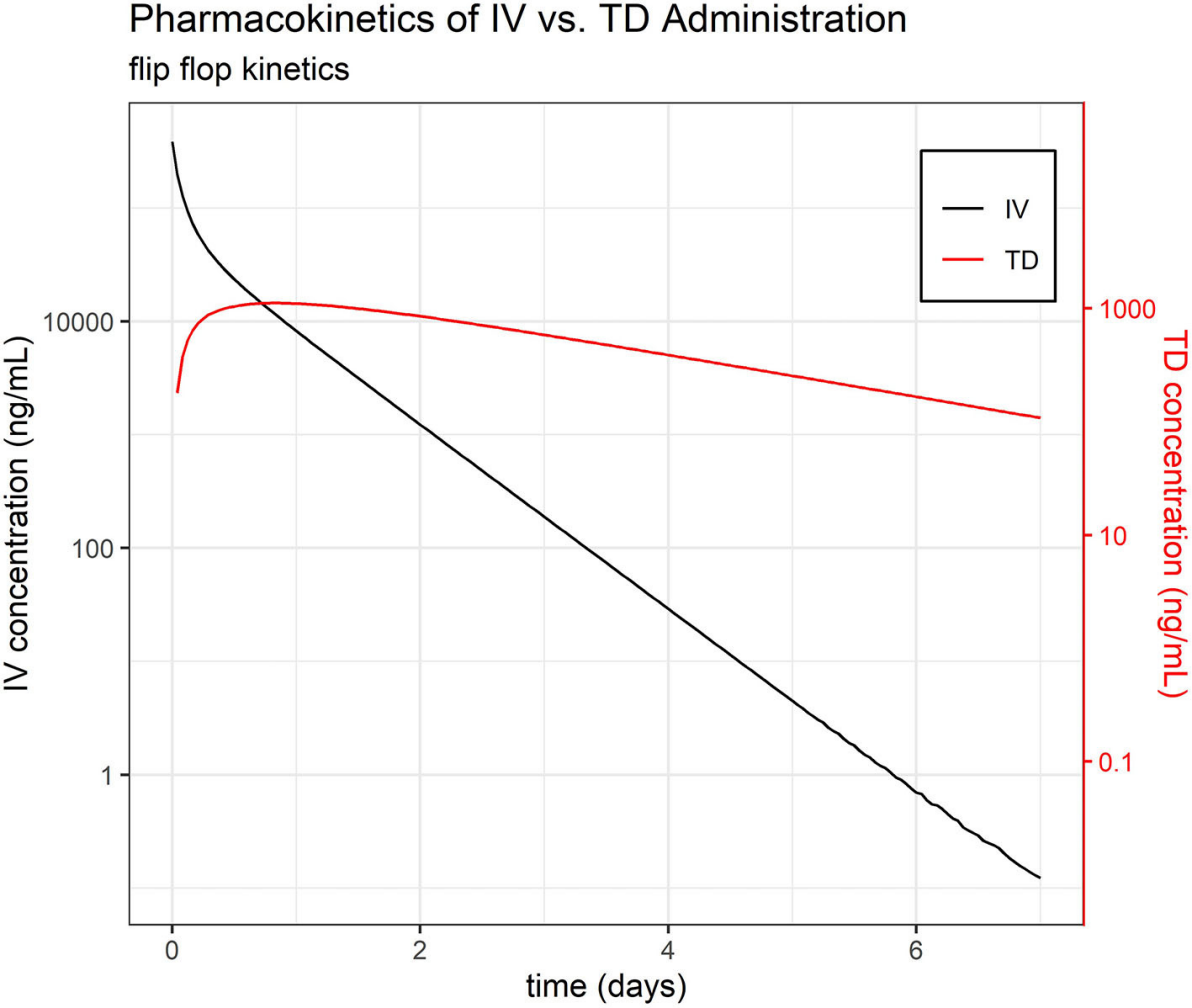
Pharmacokinetics of IV vs. TD FM administration. Average time course of FM separated by administration group, IV and TD.

Default settings in PKanalix were used, as modification was not necessary to achieve a high quality NCA. The full set of NCA PK parameters are reported in Table 1. The mean t½ were estimated at 7.06, 9.12, 11.38, and 38.89 h for IV, IM, PO, and TD, respectively—suggesting flip-flop PK for the TD route (Figure 1, Supplemental Image 1). The mean AUC_INF_/Dose were estimated at 3.4, 3.79, 3.09, and 0.20 h/L for IV, IM, PO, and TD, respectively. Evaluation of the extrapolated AUC (AUC_t−∞_) for phase I revealed up to 60% AUC_t−∞_.

**Table 1:** Noncompartmental analysis parameter table.

**Group**	**Value**	**AUC/Dose**	**AUC_**INF**_**	**Cl/F**	**Cl**	**C_max_**	**t_1/2_**	**λ_z_**	**MRT**	**t_max_**	**V_**SS**_/F**	**V_**SS**_**
	**Units**	**h/L**	mg*h/L	**L/h**	**L/h**	**mg**	**h**	**1/h**	**h**	**h**	**L**	**L**
IV	Mean	3.40	30.44	NA	0.54	12.03	7.06	0.108	5.40	0.46	NA	2.9
	SD	2.57	16.95	NA	0.42	5.49	2.10	0.036	1.74	0.79	NA	2.3
	Median	2.61	22.32	NA	0.48	11.65	6.70	0.102	5.29	0.08	NA	2.2
IM	Mean	3.79	29.19	0.3	NA	6.44	9.12	0.078	8.60	0.54	2.6	NA
	SD	1.21	10.73	0.06	NA	1.46	2.69	0.016	1.31	0.27	0.4	NA
	Median	3.52	27.55	0.3	NA	6.54	8.26	0.084	8.54	0.50	2.4	NA
PO	Mean	3.09	31.01	0.42	NA	4.99	11.38	0.060	8.33	0.93	3.5	NA
	SD	1.18	10.90	0.24	NA	1.72	1.34	0.007	1.31	0.55	1.7	NA
	Median	3.30	32.28	0.3	NA	4.88	11.36	0.060	8.59	1.00	2.7	NA
TD	Mean	0.20	2.46	9.12	NA	0.04	38.89	0.018	66.08	22.50	602.7	NA
	SD	0.14	1.29	7.44	NA	0.02	10.69	0.005	14.29	13.70	432.4	NA
	Median	0.18	2.09	5.46	NA	0.03	36.06	0.018	63.22	24.00	434.4	NA

*Several PK parameters were produced via NCA using Pkanalix2019R1 (Lixoft). The IM bioavailability and absorption rater were close to 100% and exceedingly large parameter value, respectively. Consequently the IM bioavailability was set to 100% and the absorption lag was set to 0 to stabilize the parameterization of the model. The interindividual variabilities on these fixed parameters were not estimated. The following population summary as well as dosing group summary parameters are reported: area under plasma concentration-tim curve/dose (AUC/Dose), (AUC_INF_), apparent clearance (Cl/F), clearance (Cl), maximum concentration (C_max_), terminal half-life (t_1/2_), terminal phase elimination rate constant (λ_z_), mean residence time (MRT), time of maximum concentration (t_max_), apparent volume of distribution at steady-state (V_ss_/F), and volume of distribution at steady-state (V_ss_). Mean, median and standard deviation are reported in the summary table*.

On an important note, all NCA PK parameters agree well with their NLME counterparts, further supporting the quality of the NLME model fit.

### Pharmacokinetic Model

A total of 992 plasma concentration samples of FM from IV, IM, TD, and PO dosing groups were pooled together and simultaneously modeled using NLME. Approximately 16% (156/992) of data were found to be below the LLOQ (BLQ) of the UPLC-MS validated method.

A two-compartment PK mamillary model with first order elimination was found to best describe the PK of FM in piglets. One transition compartment was used to model the PO absorption and a second parallel transition compartment was used to model the topical absorption of FM in the central compartment. The residual error was modeled as a proportional term in the mathematical model.

Sex and bodyweight did not meet the threshold for model inclusion. The metabolism rate classifier (MET) had a significant effect (*p* < 0.05) on clearance and was included in the final PK model. Correlations between clearance, intercompartmental clearance, peripheral volume, and topical bioavailability met the threshold for statistical significance and were included in the model. Of note, the MET was independent of the route of FM administration.

Overall, 9 piglets were classified as fast metabolizer (MET = 0) and 29 piglets were classified as slow metabolizer (MET = 1).

### NLME Parameter Estimates

Final parameter estimates, RSEs, and coefficients of variation can be found in Table 2. The precision of the PK parameter estimates from the final NLME model was high (RSE ≤ 25%, median RSE = 11.65%), reflecting an accurate and stable parameterization of the model. In summary, the fast metabolizer group had a clearance of 11.5 L/min, while the slow metabolizer group had a lower clearance, 4.7 L/min. The estimated volume of distribution was 1.35 L and 1.18 L for the volume of the central and the peripheral compartment, respectively.

**Table 2:** Model parameter table.

**Parameter**	**Symbol**	**Unit**	**Estimate**	**SE**	**RSE (%)**	**CV (%)**
Clearance	Cl	L/min	0.012	0.0008	7.02	31.9
Covariate Effect on Clearance	β_Cl_MET_	-	−0.892	0.07	7.84	-
Central Volume	V_c_	L	1.35	0.164	12.1	83.8
Intercompartmental Cl	Q	L/min	0.003	0.0007	22.7	227
Peripheral Volume	V_p_	L	1.18	0.169	14.3	106
Topical Bioavailability	F_top_	%	7.84	0.0168	21.5	192
Topical Absorption Rate	k_top_	1/min	0.0003	1.97E-05	7.24	29.9
Topical Absorption Lag	Lag	min	26.4	3.2	12.1	148
Oral Bioavailability	F_po_	%	100	-	-	5.62E-10
Oral Absorption Rate	k_po_	1/min	0.060	0.0101	16.7	30.5
Proportional Error	b_flunixin_	-	0.294	0.0085	2.89	-
**Correlation**		**Unit**	**Estimate**	**SE**	**RSE (%)**	**-**
corr(F_top_, Cl)		-	0.853	0.0619	7.26	-
corr(Q, Cl)		-	0.612	0.118	19.3	-
corr(Q, F_top_)		-	0.766	0.0859	11.2	-
corr(V_p_, Cl)		-	0.694	0.0962	13.9	-
corr(V_p_, F_top_)		-	0.817	0.0696	8.52	-
corr(V_p_, Q)		-	0.967	0.014	1.45	-

*Table of parameter estimates and their respective standard error estimates (SE), relative standard error estimates (RSE), and coefficients of variation (CV). The estimated steady-state volume (V_ss_) (V_ss_ = V_c_ + V_p_) is calculated as 2.53 L. During model fit, there was strong evidence of two pig subpopulations—fast metabolizers and slow metabolizers. A covariate effect on clearance was modeled to distinguish between these groups. The model of the covariate effect was ln(Cl_obs_) = ln(Cl_pred_) + β_Cl < uscore>MET_. There was no individual error on the covariate modeled, so coefficient of variation did not apply. CV of logit-normally distributed parameters was estimated by bootstrapping the conditional distribution of the parameters (n = 10,000 samples). PO bioavailability was consistently estimated at a value of ~1. To improve parameter search stability, this parameter was therefore fixed to 1 during the final model fit*.

The absolute bioavailability of PO FM (F_po_) was consistently estimated at >99% with very little inter-individual variability (IIV). Therefore, F_po_ was set to 1 and IIV_Fpo_ was set to 0.01 in the final model fit. Similarly, F_im_ was consistently estimated with little variation and a high percentage (individual F_im_ >> 99%). Therefore, it was not necessary to include F_im_ as a parameter in the final model—equivalent to F_im_ = 100% and IIV_Fim_ = 0. In contrast, the relative bioavailability of TD FM was estimated to be low (7.86%).

Overall, standard goodness-of-fit diagnostic plots—observations vs. predictions, individual fits, and prediction distribution—indicate the model reproduced the observed individual and population dynamics with high accuracy (Figures 2–4, Supplemental Images 2–9).

**Figure 2:**
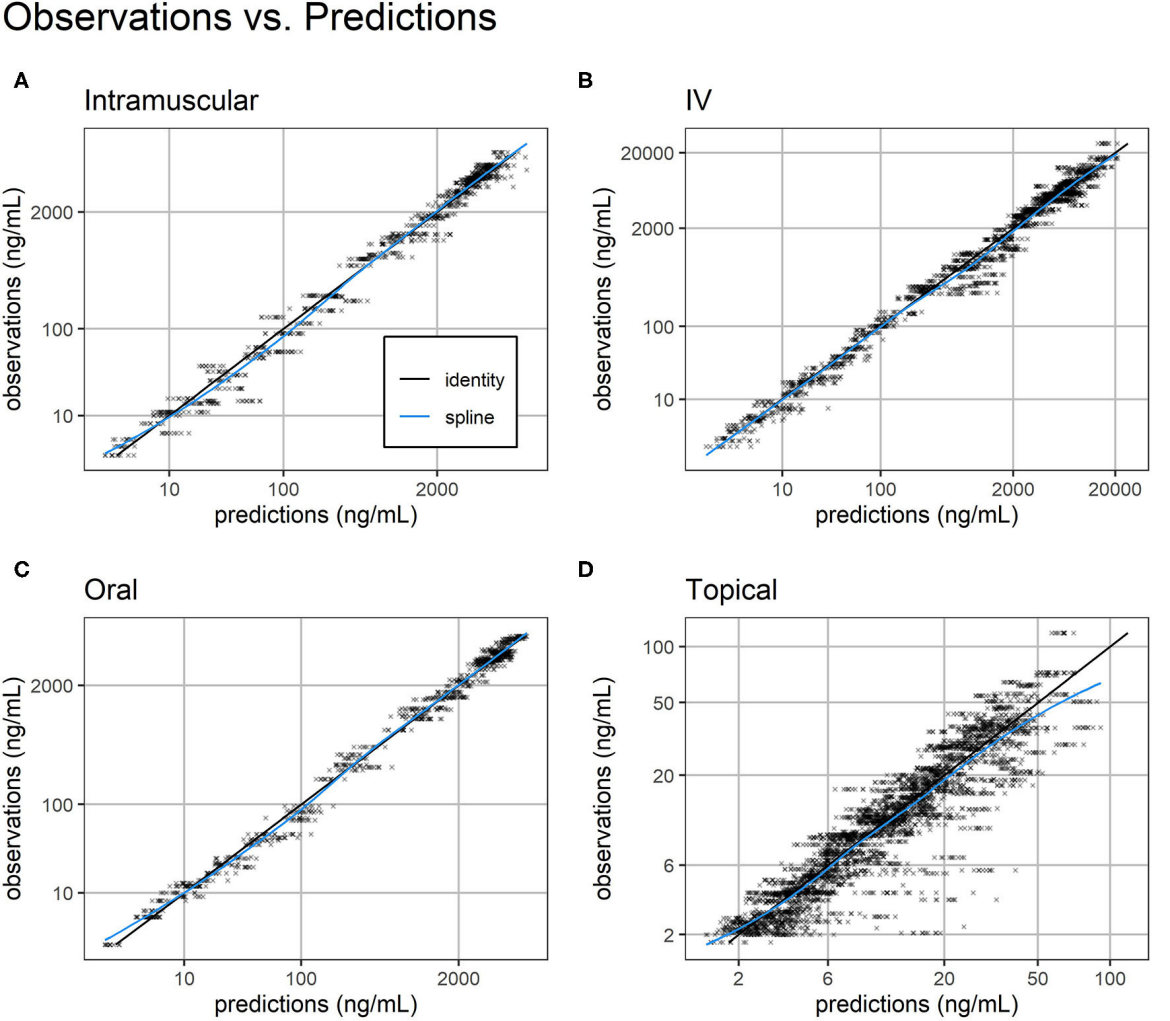
Observations vs. predictions. Observations vs. model predictions of FM concentration (black crosses) for intramuscular **(A)**, intravenous **(B)**, oral **(C)**, and topical **(D)** routes of administration. As suggested in (16), simulated individual predictions drawn from the full conditional distribution of individual predictions were used to reduce shrinkage. Plasma concentration axes were scaled by ln (“plasma concentration” + 1) to improve resolution of plasma concentrations close to zero (19). Points are semi-transparent to reduce over-plotting. The identity line (black line) and spline of the observations vs. predictions (blue line) are added to help diagnose structural misspecification.

**Figure 3:**
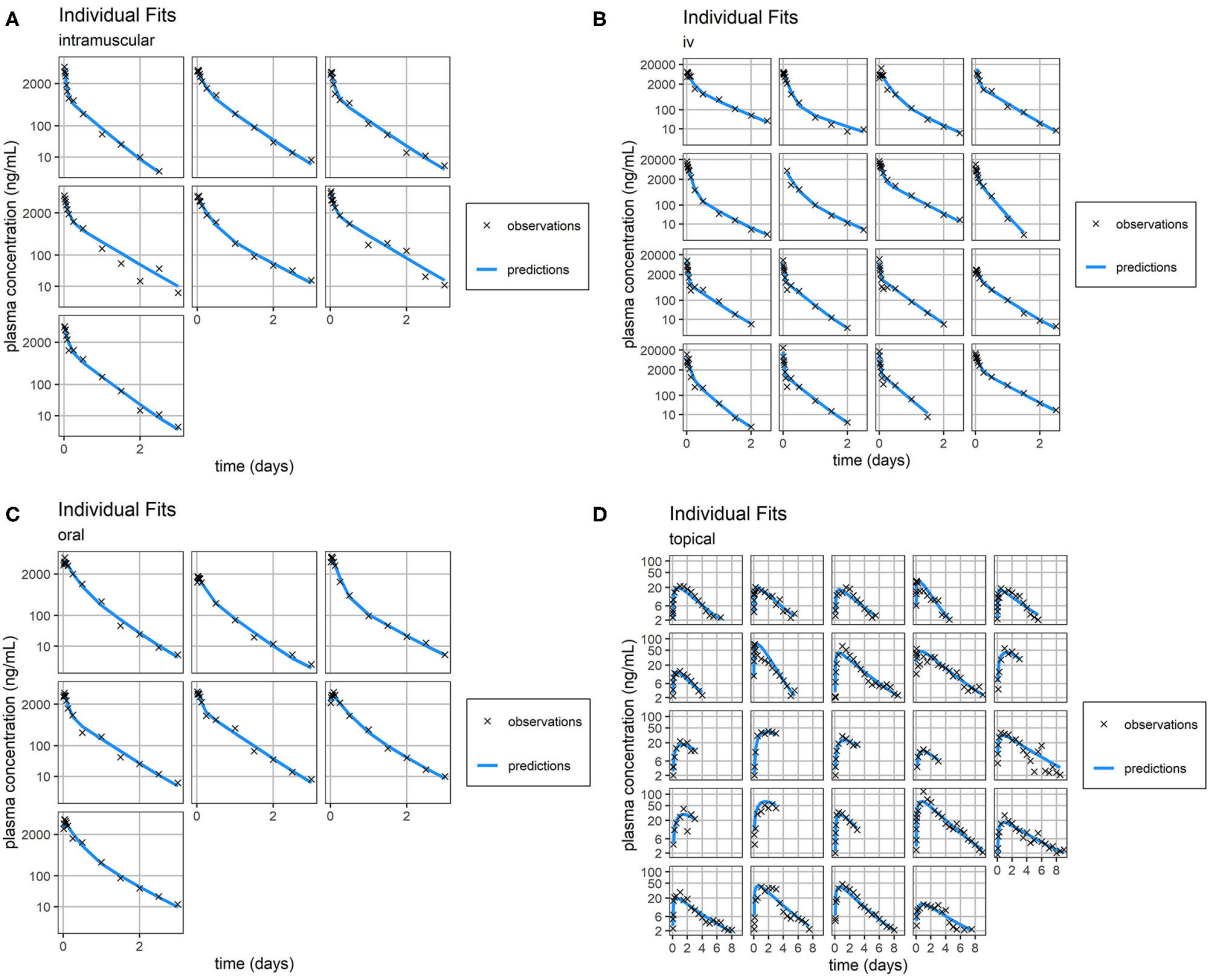
Individual Fit. Individual predictions of FM concentration time-course (blue line) vs. individual observations of FM concentration time-course (black crosses) for intramuscular **(A)**, intravenous **(B)**, oral **(C)**, and topical **(D)** routes of administration. Concentrations of FM after topical administration are relatively small. Consequently, the scale of error relative to measurement is large. Plasma concentration axes were scaled by ln (“plasma concentration” + 1) to improve resolution of plasma concentrations close to zero, as previously described (19).

**Figure 4:**
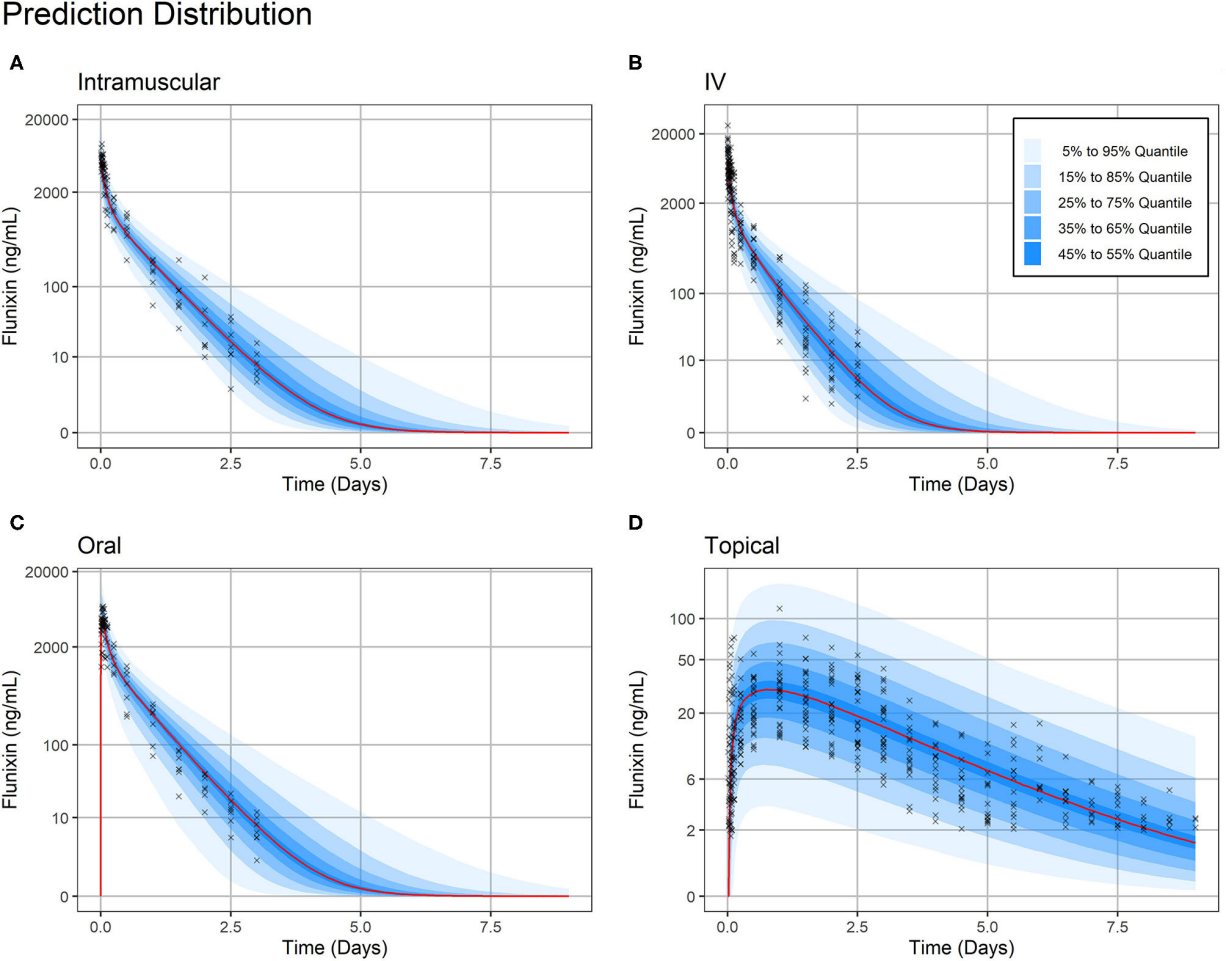
Prediction Distribution. Distribution of model predictions plotted alongside observations for intravenous **(A)**, intramuscular **(B)**, oral **(C)**, and topical **(D)** routes of administration. The prediction distribution (blue areas) was generated by Monte Carlo simulations, in this case by simulating the experiment 500 times. Then, the set of quantiles from 5% to 95% in steps of 10% {5%, 15%,…, 95} were calculated at each predicted timepoint—the average distance between predicted timepoints was 50 min (Monolix2019R1 defaults). Observations are indicated by black crosses and median prediction is indicated by a red line. Observations fall mostly within the 95% prediction interval. Plasma concentration axes were scaled by ln (“plasma concentration” + 1) to improve resolution of plasma concentrations close to zero, as previously described (19). Points are semi-transparent to reduce over-plotting.

**Figure 5:**
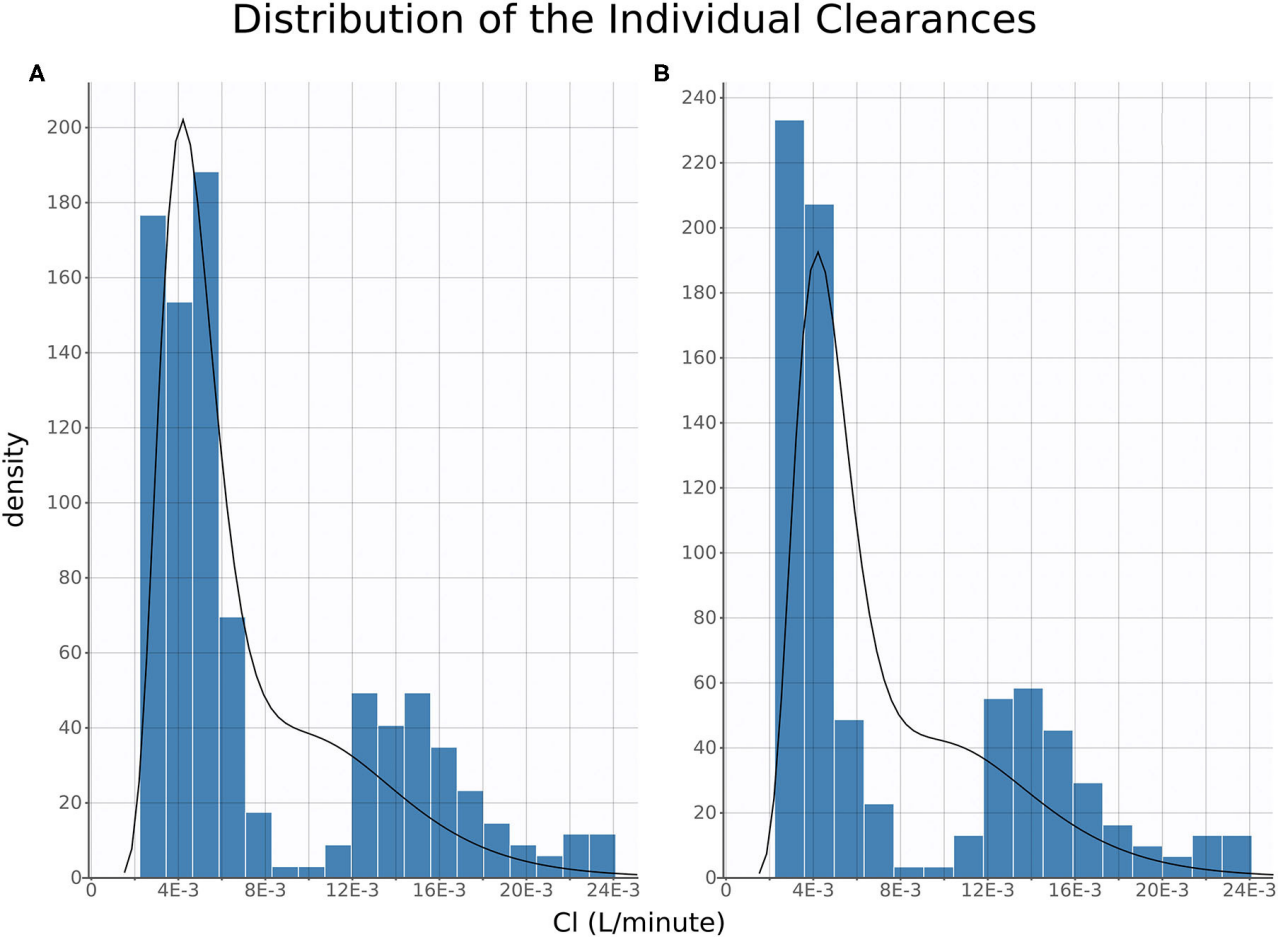
Clearance Distribution. Histograms of distribution of estimated individual clearances plotted for oral, intravenous, and intramuscular **(A)** and topical **(B)**. The histograms are produced from the full posterior distribution of individual clearances.

Of note, distribution of FM systemic clearance estimates from the final NLME model showed a homogeneous repartition of fast and slow metabolizers between TD- vs. IV/IM/PO-treated piglets (Figure 5).

### NLME Model Predictions

A sub-model of bodyweight was built for simulating individual treatments because dosages were administered in units of mg/kg. To establish this model, we pooled all sample bodyweights together and fit a *Box Cox* model of bodyweight via analysis of variance (ANOVA) in R 3.6.1 (The R Foundation for Statistical Computing) (20). The model (Equation 4) predicts a *Box Cox* transformation of bodyweight for each individual, *i*, using one parameter for population bodyweight (*BW*_*pop*_= 1.31), one parameter for inter-phase population weight variability (*IPV* = 0.89), and one residual value for each individual ($\varepsilon_{i}\;\sim \;N\left(0,0.29^{2}\;\right)$.

Equation 4:

$$\begin{array}{l} \frac{BW_{i}^{\lambda }-1}{\lambda }\;=\;BW_{pop}\;+\;IPV\;+\;\varepsilon_{i}\\ \varepsilon_{i}\;\sim \;N\left(0,\sigma_{BW}^{2}\right) \end{array}$$

The optimal λ (λ = 0.14) was selected by trying values from−2 to 2 in steps 0.1, and determining which λ minimized sum of squared error (i.e., $\sum {\varepsilon_{i}}^{2}$) in a spline interpolation curve between points.

**Figure 6:**
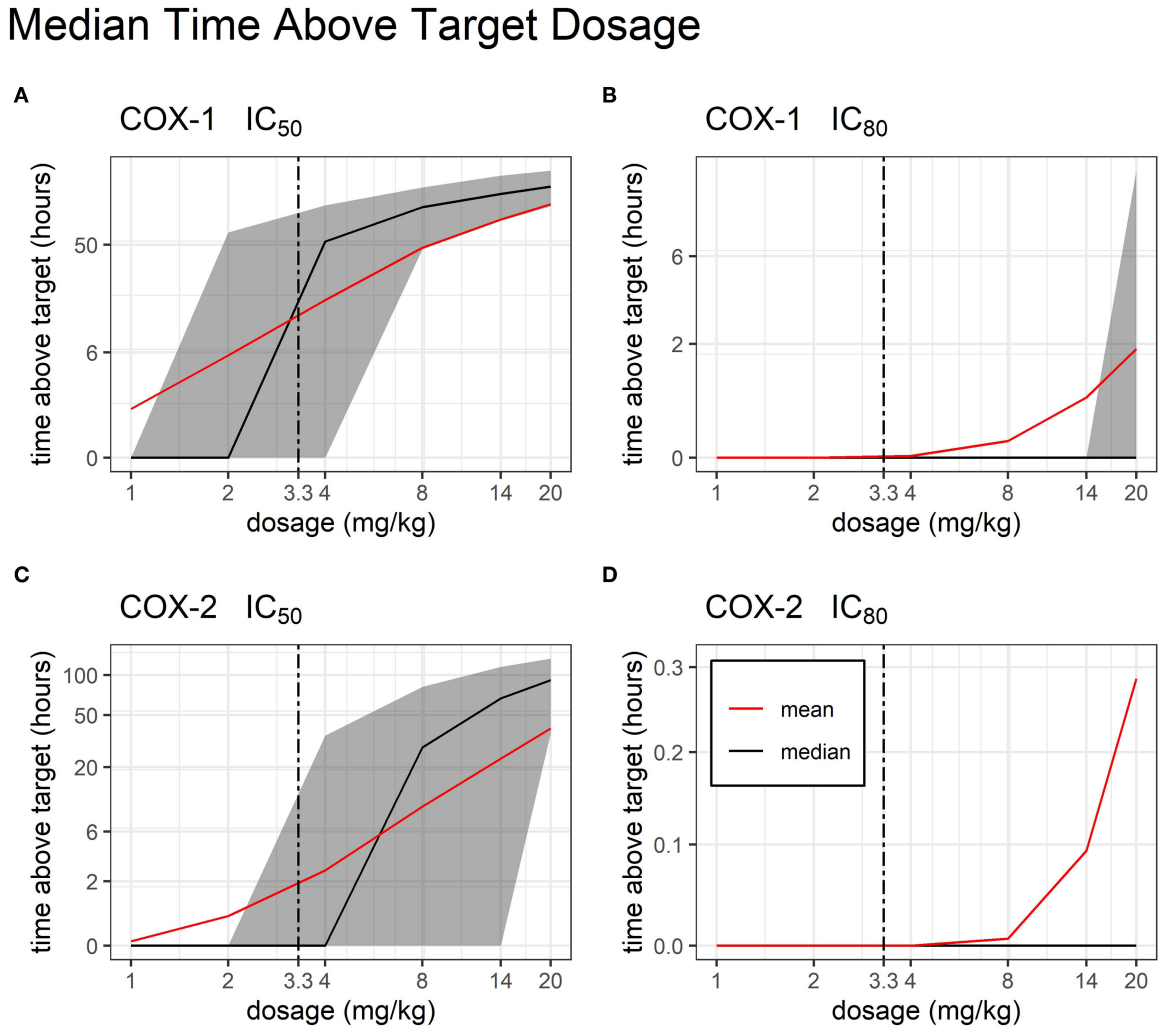
Time Above Target by Dosage. Median and mean (black and red line, respectively) time duration (hours) for which FM plasma concentrations stay above target COX inhibition following single administration of a TD dose of FM (from 1 to 20 mg/kg). COX-1 IC_50_ **(A)**, COX-1 IC_80_ **(B)**, COX-2 IC_50_ **(C)**, and COX-2 IC_80_ **(D)** are represented. Interindividual variability in target attainment is represented via the filled areas surrounding each median. The areas span the 50% interquartile range between the 25th and 75th percentile. The time above target axis was scaled by ln(“time above target” + 1) to improve resolution of time values close to or equal to zero, as previously described (19). The axis for time was scaled by log2.

After building a sub-model of bodyweight, we were able to simulate the total time above target concentrations at 1, 2, 4, 8, 14, and 20 mg/kg dosage (Figure 6). We found that for COX-1 IC_50_ and COX-2 IC_50_, there are several viable TD dosages which produced therapeutic concentrations for >24 h with very little variability in time above target.

## Discussion

Previous researchers have evaluated the PK of FM after IV, IM, and PO in mature swine (21, 22), while FM PK in piglets has been evaluated for the IV and IM route of administration (23–26). Additional research for TD in mature swine has also been reported since the completion of this study (27). Although the PK of IV FM has been reported in piglets (24), the IV route was studied here to establish the absolute bioavailability of FM for extravascular routes of administration. To the best of the authors' knowledge, the PK evaluation of IM, PO, and TD routes of administration of FM in pre-wean piglets has not been reported. The objective of this study was to establish the PK of IV, IM, PO, and TD administration of FM and evaluate the ability of TD FM to reach therapeutic levels in pre-wean piglets.

Nonlinear mixed-effects (NLME) modeling is routinely used in human pharmaceutical research (28), and veterinary scientists are increasingly applying NLME techniques to address animal health related issues (13). Individual animal characteristics (covariates), such as age, sex, and GI absorption can influence drug disposition kinetics. Additionally, NLME modeling allows individual animal covariates to be analyzed to identify population characteristics that impact drug PK (13). The use of NLME modeling in this study quantified individual variability by accounting for the following covariates: age, sex, and drug metabolism. NLME also provided a means for pooling IV, IM, PO, and TD administration data to provide an analytical framework for the simultaneous modeling of 992 concentrations time points. In addition to NLME modeling, this study also provided drug exposure after administration (AUC) and PK parameters using noncompartmental analysis (NCA). The use of NCA combined with NLME provides verification of the models used and the results provided. Our calculated V_ss_ using the NLME approach was 2.53L (Table 2) and compares favorably to the estimated median of V_ss_ from the NCA approach in Table 1 (2.3 L). The fact that our calculated V_ss_ is slightly larger than the reported median V_ss_ is consistent with previous literature (29). Additionally, our estimated V_ss_ is consistent with previously reported values in swine (23). The heritability of the pigs was not within the scope of this project, however, a previous study has shown that heritability can have an effect on FM PK parameters, which may explain the two drug metabolism groups (30). Additionally, the stress that can be induced by repeated blood collections, individual housing, and early weaning could contribute to differences in drug metabolism (31).

The target dose of 2.2 mg/kg for IM administration is according to the label and the same target dose was used for administration of IV dose (32). The C_max_ for PO administration in this study is higher than previous studies in which PO PK parameters were evaluated for mature swine (22). The high C_max_ for PO administration in this study is comparable to peak concentrations after IM administration. The t_max_ for PO administration in this study is consistent with t_max_ achieved in mature swine (22). The target dose of 3.3 mg/kg for TD route followed the label of the commercial product for administration in cattle (33). The blood collection time points and washout period for TD application in this study were acquired from PK research conducted in cattle (8). Transdermal FM produced systemic drug levels consistent with concentrations in sows (27). However, there appears to be no additional research on TD administration of FM in piglets for comparison. The t_max_ for TD administration achieved in this study is longer than previous research in sows (27). Absolute bioavailability quantifies the fraction of a drug that is absorbed and available to produce its systemic effect (34). Estimated absolute bioavailability of >99% for the PO route of administration is higher than previously reported in mature swine. Although the bioavailability for the TD route was higher than an earlier description in mature swine (27), there is insufficient evidence to conclude that the TD route could provide pain mitigation. One explanation for the higher PO bioavailability in this study may be the formulation and administration technique. An orogastric tube was used to orally administer the injectable formulation of FM to ensure that the full dose was administered to the stomach, while a previous study used a powdered formulation mixed with cookie dough (27). Although orogastric administration would not be considered for on-farm use, the method is optimal for precise dose administration and initial PK analysis. The low estimated *F* for the TD route could be attributed to the anatomy of the skin—with drug accumulation in intracellular spaces and overall poor diffusion to the systemic circulation through the various skin layers (35). The low estimated concentration of FM after TD dosing, concurrent with poor absolute bioavailability, is not expected to mitigate pain in pre-wean piglets in the conditions of this study, which included individual housing of the piglets. Flunixin meglumine is described as a weak acid with approximately 99% plasma protein binding (Banamine®-S, Merck Animal Health, Madison, NJ, USA). The high degree of plasma binding along with the low pKa (5.82) can contribute to the low volume of distribution, which also suggests minimal tissue binding. FM has a low extraction ratio, which will allow the free drug concentration to be unchanged independent of the unbound drug, which could account for the high clearance (36, 37).

The phenomenon of flip-flop PK in extravascular dosing routes occurs when absorption (instead of elimination) becomes the rate limiting step in drug PK. Comparison of the estimated slope of the terminal phase (and their associated elimination half-life, t½) between intravascular and extravascular dosing can indicate the presence of flip-flop PK. While the average t½ of FM after IV, IM, and PO dosing was consistently estimated at 0.28, 0.34, and 0.47 days, respectively, the average elimination half-life of FM after TD dosing was much longer at 1.50 days, which is highly suggestive of flip-flop PK (35).

Research on NSAIDs has shown that the ability to achieve a therapeutic effect is dependent upon the ability to achieve at least 80% inhibition (IC_80_) of COX-2. As a non-selective COX inhibitor, FM also inhibits COX-1, which is associated with unwanted gastrointestinal toxicities such as intestinal erosions and ulceration (18, 38–40). The authors conducted a literature search and are not aware of research that has established swine IC_50_ and IC_80_ values for the COX isoforms. Although the use of values from another species is a study limitation, it is well documented that NSAIDs cause GI toxicity due to their action on the arachidonic acid pathway; therefore, data from equine research was used in this study to extrapolate swine IC_50_ and IC_80_ to determine anti-COX activity (18). A core objective of this study was to estimate a therapeutic and safe dose for TD FM in piglets. Our Monte Carlo simulations show that increasing the FM TD dose up to 20 mg/kg provides FM concentrations above the IC_80_ target for COX-2 for <1 h. Incidentally, this very high dose would cause increased COX-1 inhibition for >2 h. Overall, these results suggest that this increased formulation of TD FM is unable to provide systemic anti-inflammatory concentration that are consistent with pain relief in pre-wean piglets.

Piglet stress induced by early weaning, individual housing, and repeated venipuncture for blood collections is a limitation of this study. Pre-weaning processing that necessitates FM would typically occur prior to weaning and there is a need for FM PK parameters in young pigs. Although the pigs were weaned early, the pigs did have adequate nutrition throughout the trial. While there is no previous research in swine, research in cattle has shown that grooming activities can lead to variable PK parameters (41). As an initial PK trial, the authors provided individual housing to decrease PK variability that may be associated with grooming activities after TD administration. A jugular catheter could reduce incidence of venous hematomas and stress associated with venipuncture, however the authors are not aware of a successful catheterization technique in pre-wean piglets. Although the TD piglets in phase II were subjected to more blood collections than TD piglets in Phase I, our initial data evaluation in phase I showed that AUC_t−∞_ was up to 60%. Additional blood collections in phase II reduced the AUC_t−∞_. Additionally, the research and blood collection parameters were approved by the Iowa State University Institutional Animal Care and Use Committee (IACUC #18-057, #18-169).

In conclusion, our study provides the very first comprehensive characterization of FM PK in pre-wean piglets. The use of NLME modeling allowed for the evaluation of all available routes of FM for a robust interpretation of the data. Future applications of NLME consist of pooling larger study populations of swine FM data for a more robust characterization of the impact of individual covariates on FM PK (ongoing work for follow-up publication). Overall, the TD route has very poor bioavailability and should not be considered an option for therapeutic use in piglets. The authors of this study are not aware of any ongoing investigation of the effects of PO or IM FM administration in pre-wean piglets. Given the high bioavailability of these administration routes, we believe this to be a worthwhile area for further research during castration and tail-docking in the US swine industry.

## Data Availability Statement

Phase II time-concentration dataset is provided as Supplemental Tables 1, 2. Additional raw data supporting the conclusions of this manuscript will be made available by the authors, without undue reservation, to any qualified researcher.

## Ethics Statement

The animal study was reviewed and approved by Institutional Animal Care and Use Committee, Iowa State University.

## Author Contributions

HK contributed to study design, sample collection and analysis, and manuscript preparation and submission. JM and BS performed the pharmacological modeling, contributed to study design, and manuscript preparation. JC contributed to the study design and the manuscript preparation. JB, AF, KH, BR, and KS contributed to study design and sample collection. SR conducted drug concentration analysis of plasma samples. LK led grant submission and contributed to study design, sample collection and analysis, and manuscript preparation. All authors have read and approved the final manuscript.

## Conflict of Interest

The authors declare that the research was conducted in the absence of any commercial or financial relationships that could be construed as a potential conflict of interest.

## Supplementary Material

Supplemental Table 1 Individual time-concentration dataset for Phase II.

Supplemental Table 2 Individual time-concentration dataset for Phase II.

Supplemental Image 1 Pharmacokinetics of IV vs. TD FM administration (0–24 h). Average time course of FM (0–25 h) separated by administration group, IV and TD.

Supplemental Image 2 Prediction Distribution (0–24 h). Distribution of model predictions plotted alongside observations for intramuscular route of administration.

Supplemental Image 3 Prediction Distribution (0–24 h). Distribution of model predictions plotted alongside observations for intravenous route of administration.

Supplemental Image 4 Prediction Distribution (0–24 h). Distribution of model predictions plotted alongside observations for oral route of administration.

Supplemental Image 5 Prediction Distribution (0–24 h). Distribution of model predictions plotted alongside observations for topical route of administration.

Supplemental Image 6 Individual Fit (0–24 h). Individual predictions of FM concentration time-course (blue line) vs. individual observations of FM concentration time-course (black crosses) for intramuscular from 0 to 24 h.

Supplemental Image 7 Individual Fit (0–24 h). Individual predictions of FM concentration time-course (blue line) vs. individual observations of FM concentration time-course (black crosses) for intravenous from 0 to 24 h.

Supplemental Image 8 Individual Fit (0–24 h). Individual predictions of FM concentration time-course (blue line) vs. individual observations of FM concentration time-course (black crosses) for oral from 0 to 24 h.

Supplemental Image 9 Individual Fit (0–24 h). Individual predictions of FM concentration time-course (blue line) vs. individual observations of FM concentration time-course (black crosses) for topical from 0 to 24 h.
