# Supplementary material for: Pharmacokinetics of Intravenous, Intramuscular, Oral, and Transdermal Administration of Flunixin Meglumine in Pre-wean Piglets
Source: Front Vet Sci. 2020 Aug 28;7:586. doi: 10.3389/fvets.2020.00586 (PMC7485418; doi:10.3389/fvets.2020.00586)
Supplement: Supplemental Table 2 — Individual time-concentration dataset for Phase II. [file Table_2.DOCX]

ID – arbitrary numerical ID assigned to piglets.

TIME – time after first occasion dose in minutes.

AMT – amount of flunixin meglumine administered in *ng*.

ODV – log of flunixin plasma concentration sample in units of *log(ng/mL).*

DV – flunixin plasma concentration sample in units of *ng/mL.*

CENS – binomial column encoding whether measurement is below limit of quantification (BLQ). 1 represents BLQ and 0 represents not BLQ.

ADM – encodes route of administration, 1 = oral; 2 = topical; 3 = intramuscular; and 4 = iv.

OCC – encodes the relative occasion of time. Each time = 0 is relative to the occasion block. Common for non-linear mixed effects modeling. This column allows for exploration of inter-occasion variability.

COV – the weight in kg of each animal.

TOP – a binomial covariate indicating whether the administration was topical, nor something else. 1 represents topical and 0 represents not topical.

MET – a categorical covariate indicating whether this piglet was a relatively slow metabolizer of flunixin where MET = 0 encodes ‘fast’ and MET = 1 encodes ‘slow.’

CAT – category encoding route. Note: during data exploration, we noticed concentration timecourse from some piglets administered flunixin meglumine via IV appeared to exhibit an absorption phase. We took note of this by naming these rows *‘ivsc’* which stands for *‘possibly combination of intravenous and subcutaneous.’*

In the dataset, the character ‘.’ represents missing or NA. See [Monolix’s web documentation](http://monolix.lixoft.com/data-and-models/creating-data-set/) for further details on dataset definition.
