# Supplementary figures and images for: Pharmacokinetics of Intravenous, Intramuscular, Oral, and Transdermal Administration of Flunixin Meglumine in Pre-wean Piglets
Source: Front Vet Sci. 2020 Aug 28;7:586. doi: 10.3389/fvets.2020.00586 (PMC7485418; doi:10.3389/fvets.2020.00586)

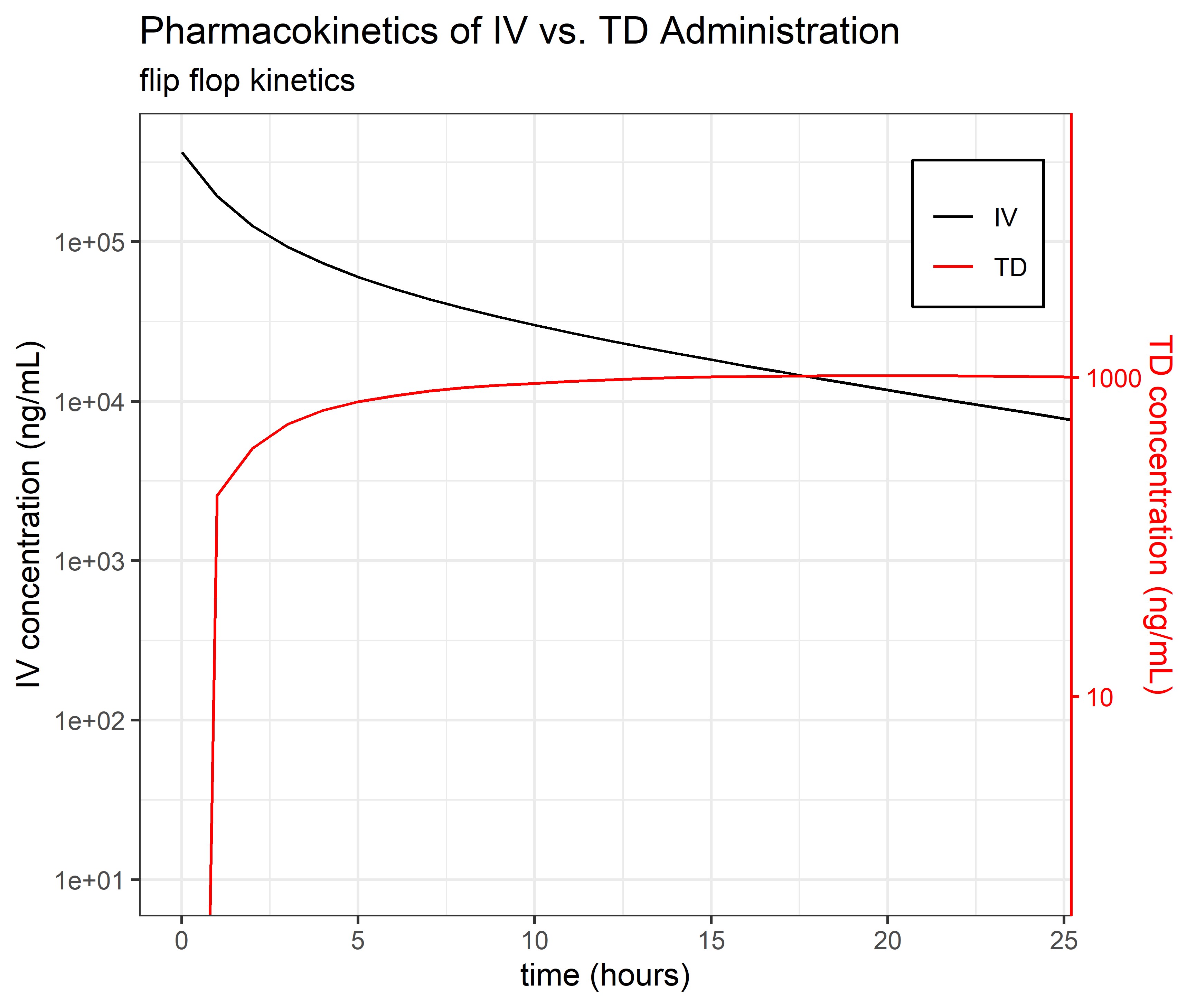

Supplement: Supplemental Image 1 — Pharmacokinetics of IV vs. TD FM administration (0–24 h). Average time course of FM (0–25 h) separated by administration group, IV and TD. [file Image_1.JPEG]

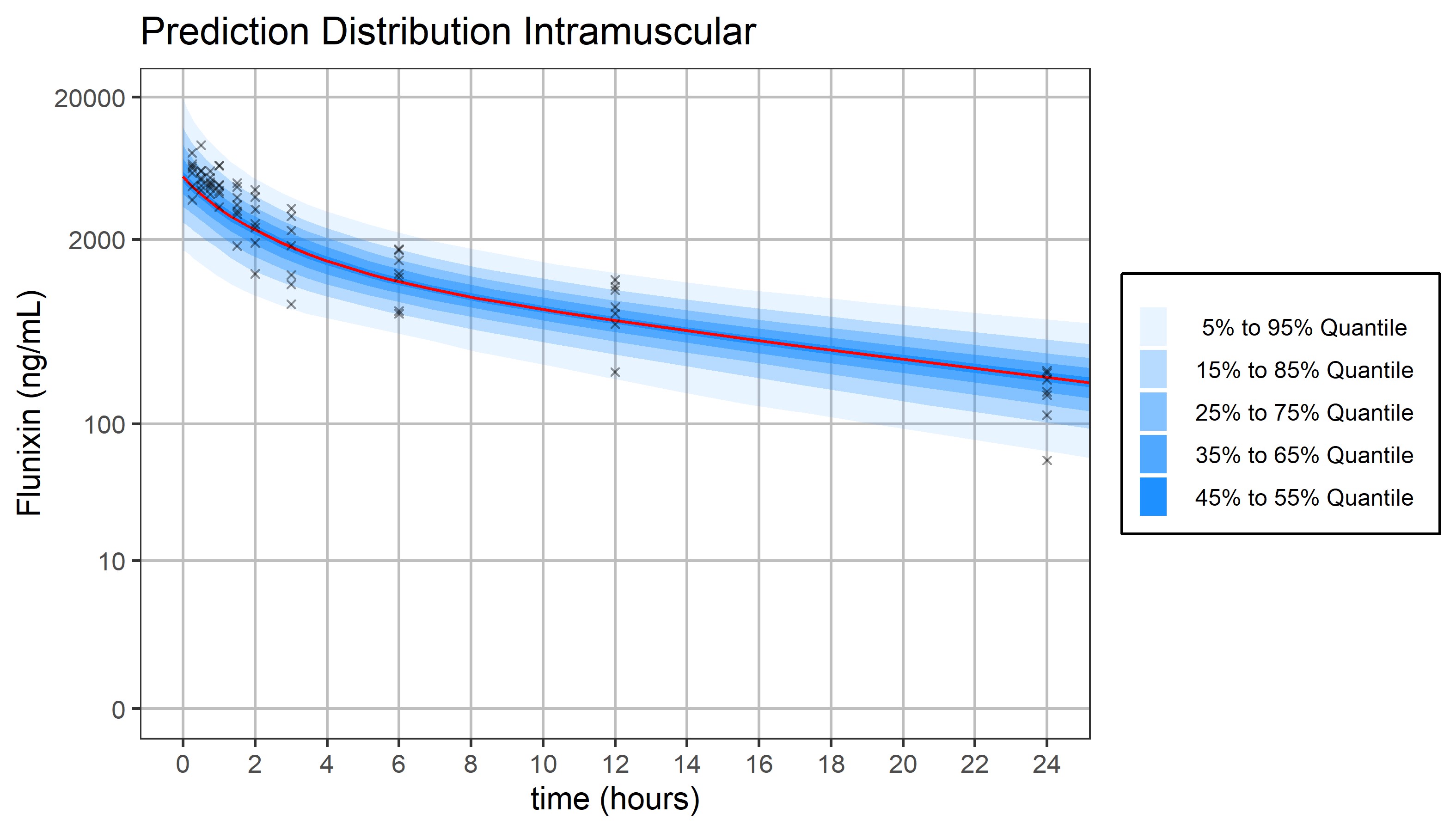

Supplement: Supplemental Image 2 — Prediction Distribution (0–24 h). Distribution of model predictions plotted alongside observations for intramuscular route of administration. [file Image_2.JPEG]

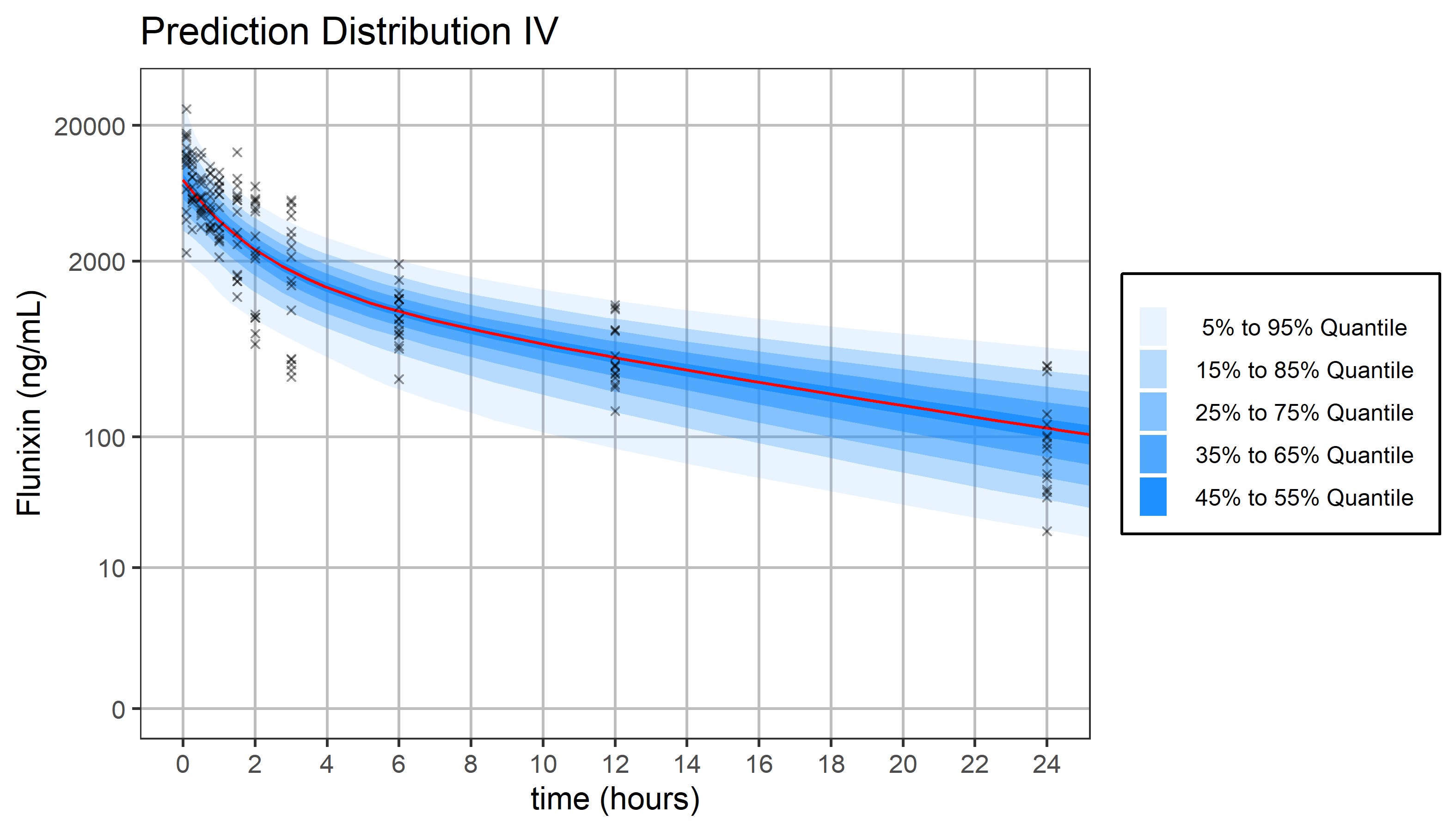

Supplement: Supplemental Image 3 — Prediction Distribution (0–24 h). Distribution of model predictions plotted alongside observations for intravenous route of administration. [file Image_3.JPEG]

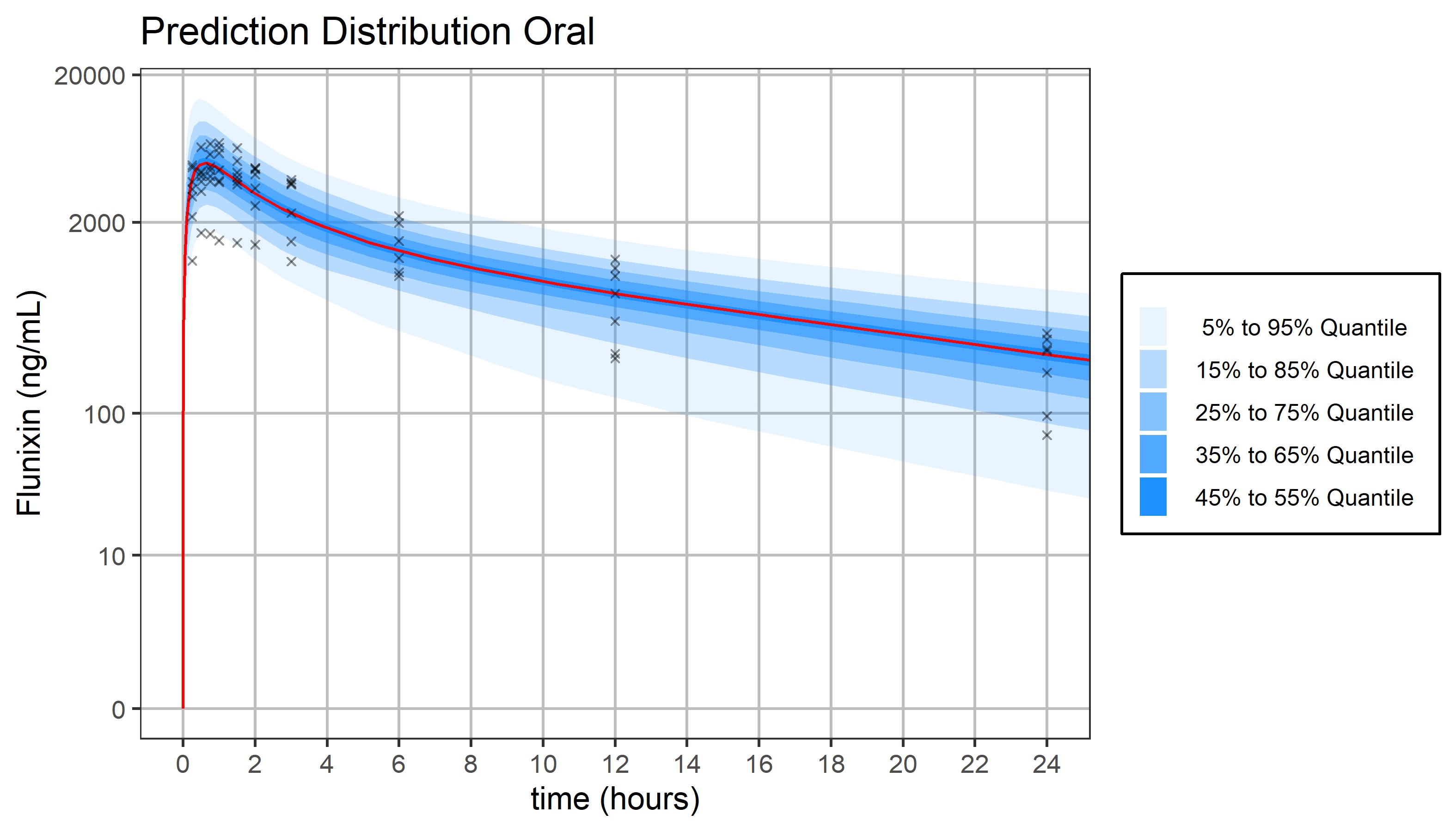

Supplement: Supplemental Image 4 — Prediction Distribution (0–24 h). Distribution of model predictions plotted alongside observations for oral route of administration. [file Image_4.JPEG]

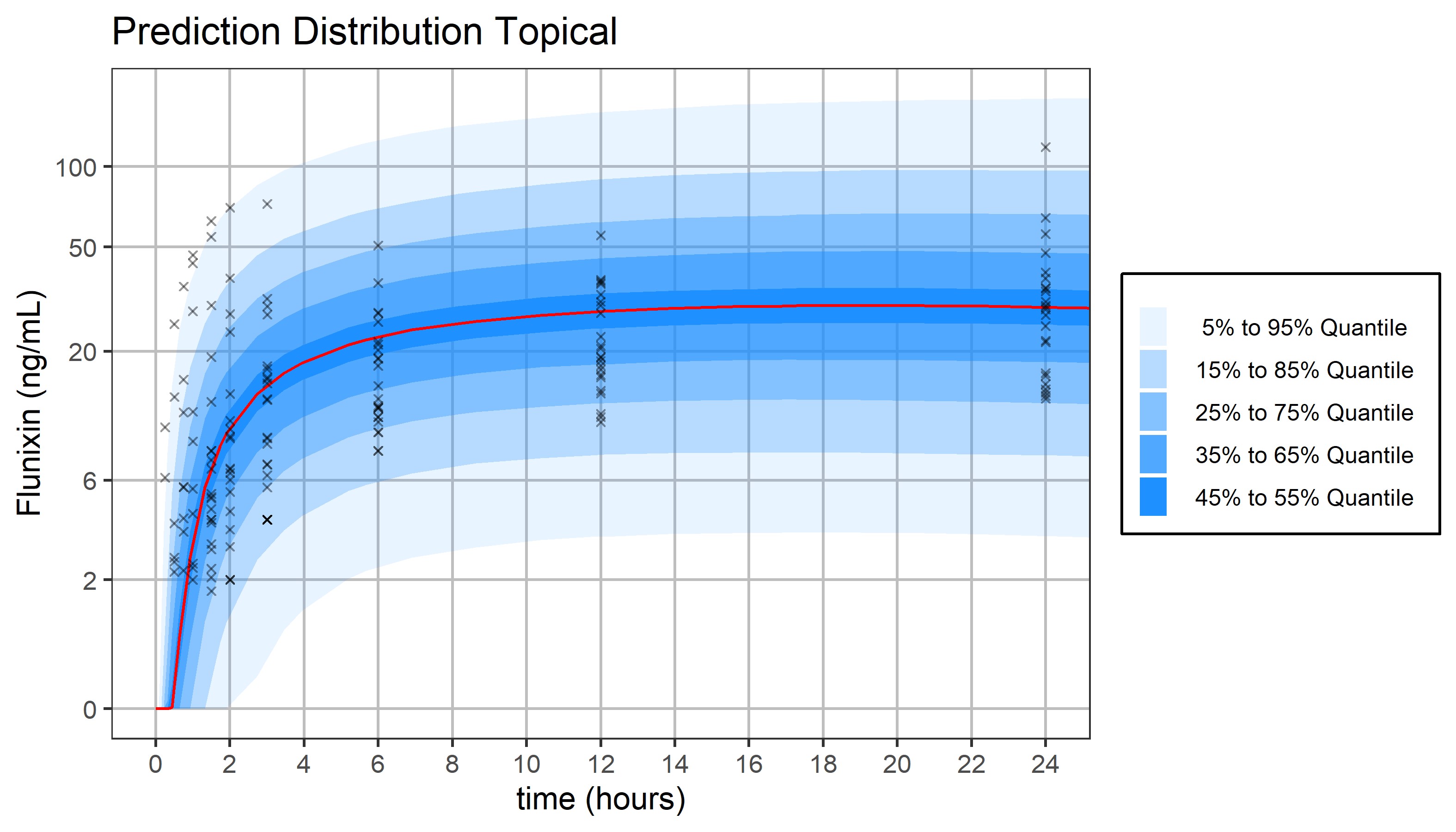

Supplement: Supplemental Image 5 — Prediction Distribution (0–24 h). Distribution of model predictions plotted alongside observations for topical route of administration. [file Image_5.JPEG]

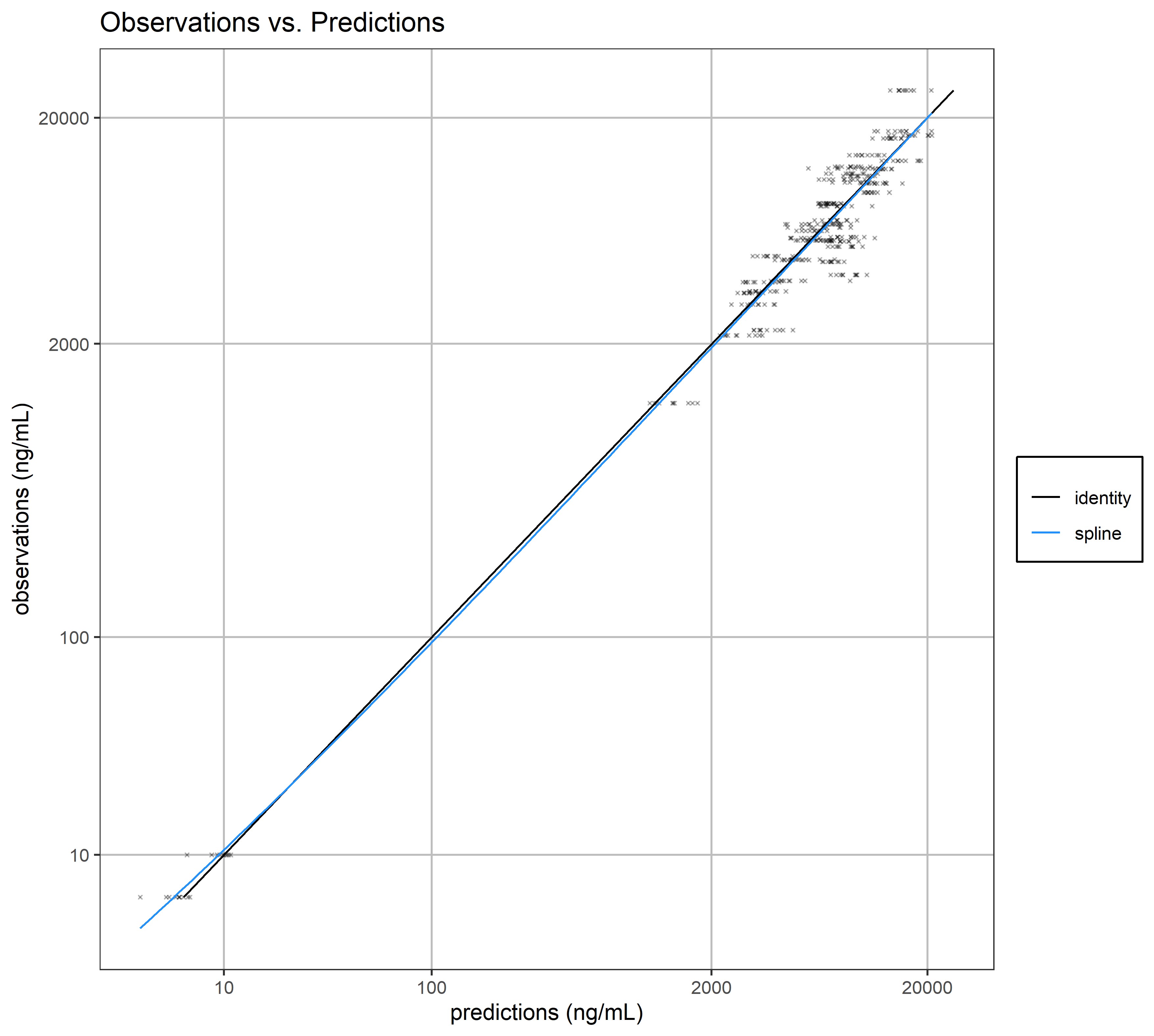

Supplement: Supplemental Image 6 — Individual Fit (0–24 h). Individual predictions of FM concentration time-course (blue line) vs. individual observations of FM concentration time-course (black crosses) for intramuscular from 0 to 24 h. [file Image_6.JPEG]

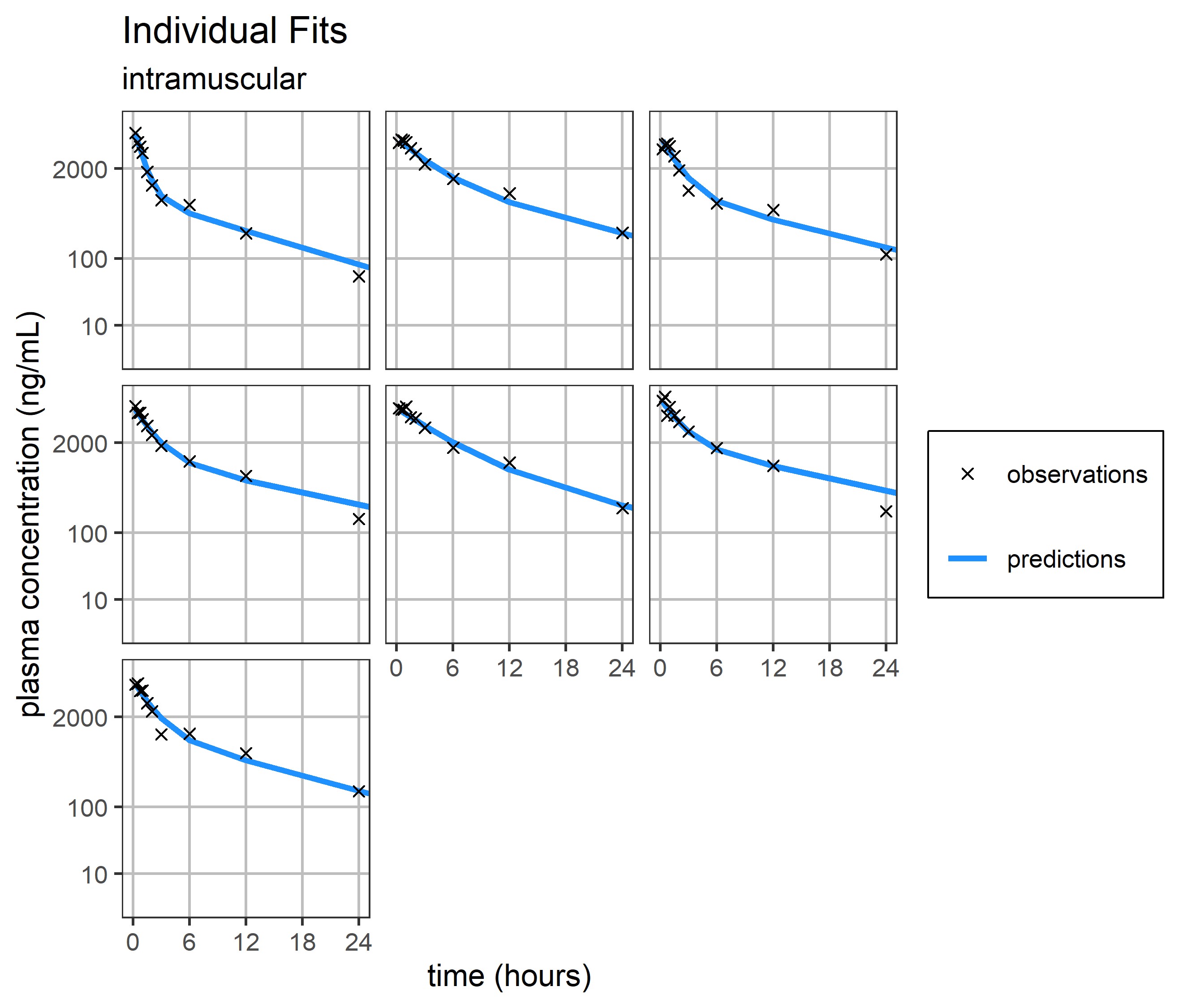

Supplement: Supplemental Image 7 — Individual Fit (0–24 h). Individual predictions of FM concentration time-course (blue line) vs. individual observations of FM concentration time-course (black crosses) for intravenous from 0 to 24 h. [file Image_7.JPEG]

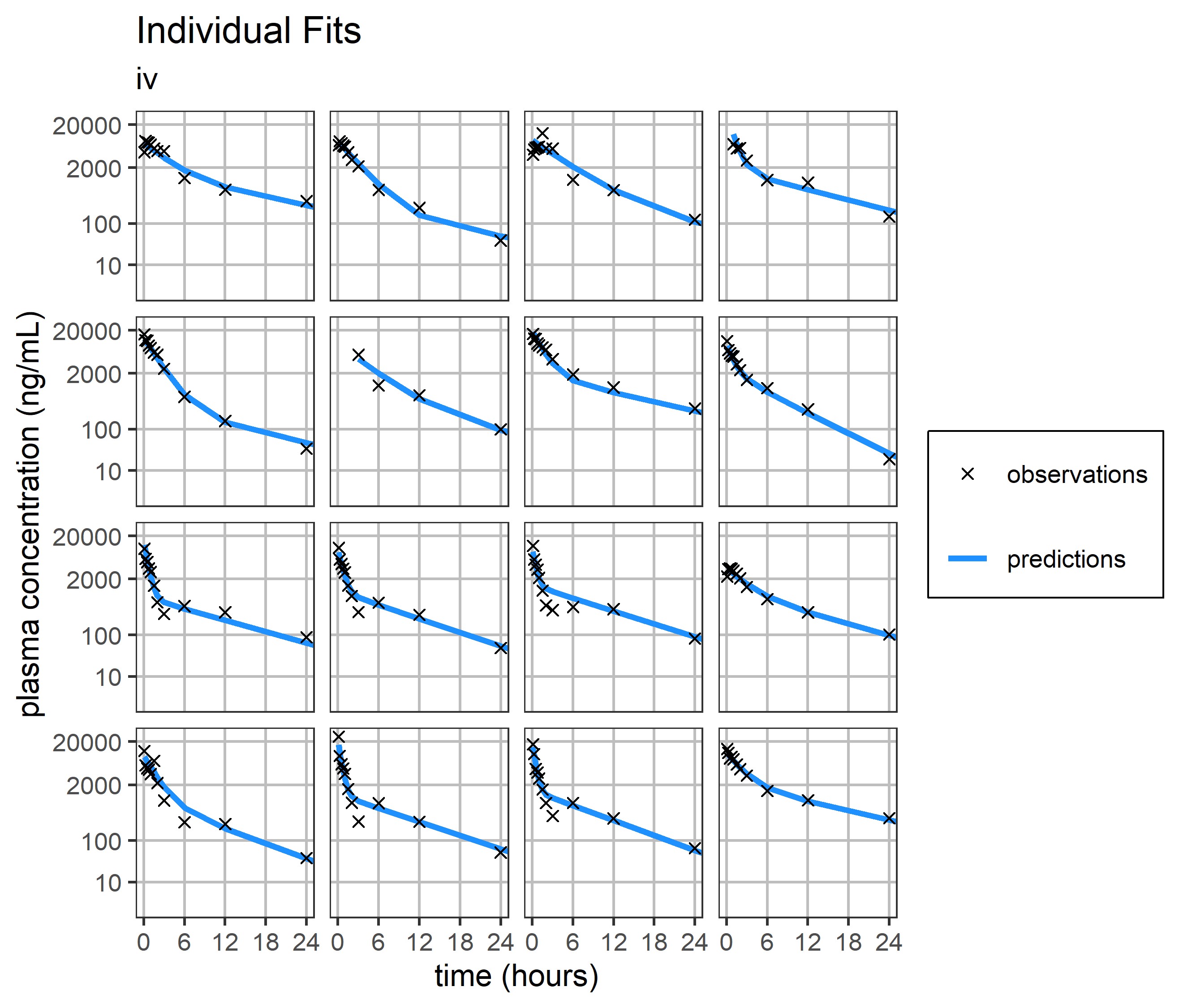

Supplement: Supplemental Image 8 — Individual Fit (0–24 h). Individual predictions of FM concentration time-course (blue line) vs. individual observations of FM concentration time-course (black crosses) for oral from 0 to 24 h. [file Image_8.JPEG]

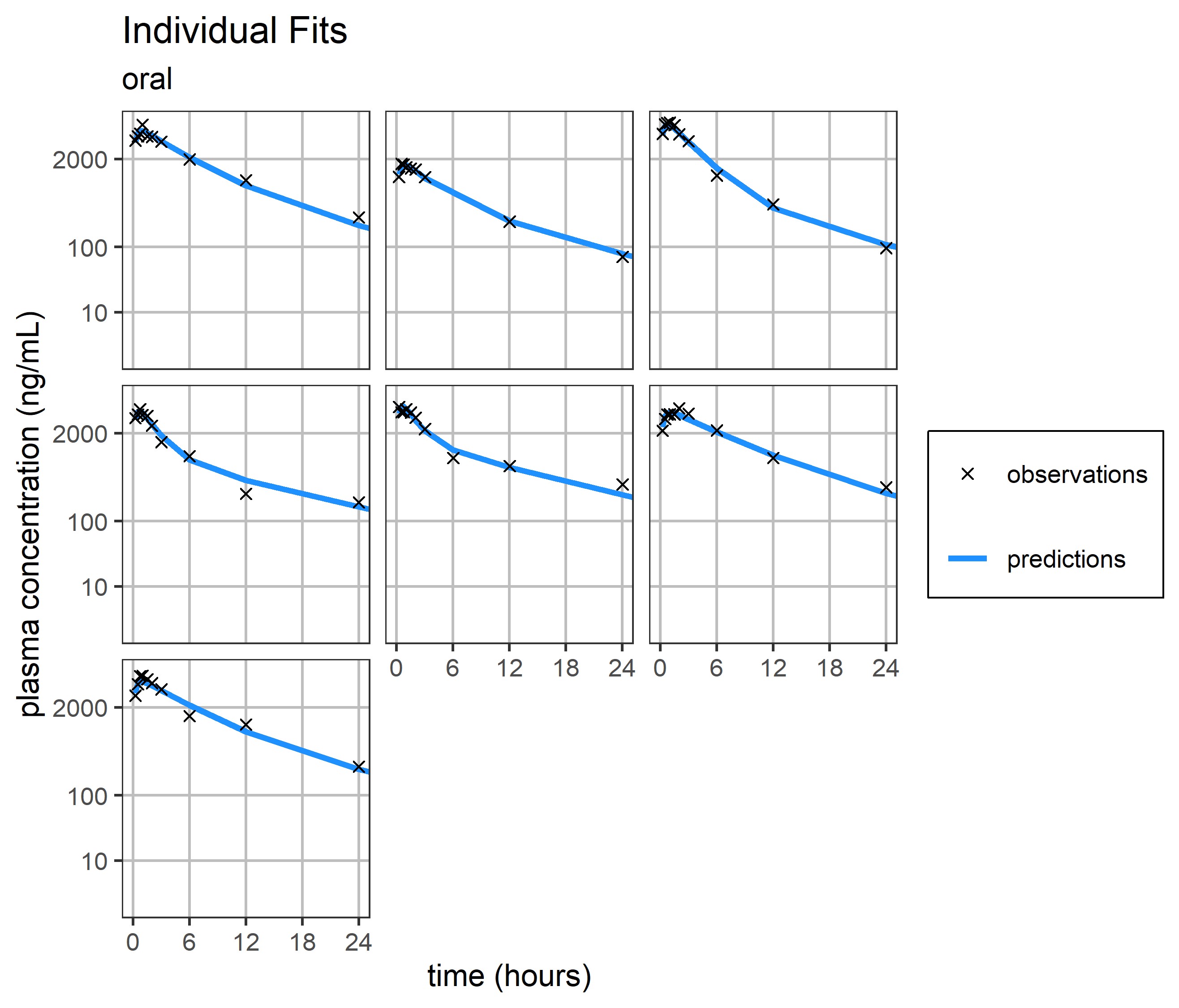

Supplement: Supplemental Image 9 — Individual Fit (0–24 h). Individual predictions of FM concentration time-course (blue line) vs. individual observations of FM concentration time-course (black crosses) for topical from 0 to 24 h. [file Image_9.JPEG]

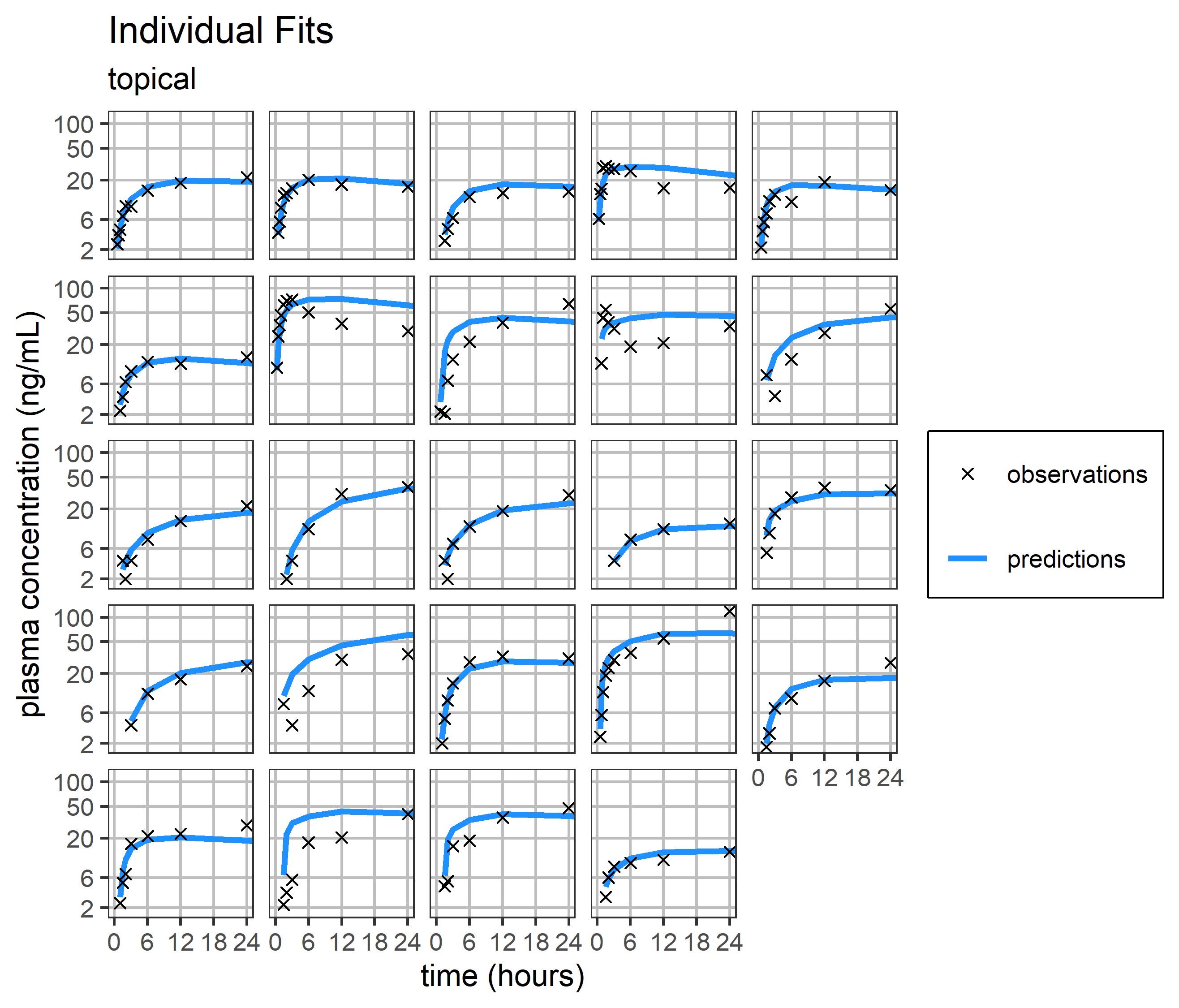

Supplement: Supplementary file 12 [file Image_10.JPEG]
